# DNA damage targeted therapy for advanced breast cancer

**DOI:** 10.37349/etat.2024.00241

**Published:** 2024-06-25

**Authors:** Vanessa Patel, Sandra Casimiro, Catarina Abreu, Tiago Barroso, Rita Teixeira de Sousa, Sofia Torres, Leonor Abreu Ribeiro, Gonçalo Nogueira-Costa, Helena Luna Pais, Conceição Pinto, Leila Costa, Luís Costa

**Affiliations:** IRCCS Istituto Romagnolo per lo Studio dei Tumori (IRST) “Dino Amadori”, Italy; ^1^Oncology Division, Unidade Local de Saúde Santa Maria, 1649-028 Lisboa, Portugal; ^2^Instituto de Medicina Molecular João Lobo Antunes, Faculdade de Medicina de Lisboa, Universidade de Lisboa, 1649-028 Lisboa, Portugal; ^3^Pharmacy Department, Unidade Local de Saúde Santa Maria, 1649-028 Lisboa, Portugal

**Keywords:** BReast CAncer 1/2 gene, breast cancer, chemotherapy, homologous recombination, poly[adenosine diphosphate-ribose] polymerase

## Abstract

Breast cancer (BC) is the most prevalent malignancy affecting women worldwide, including Portugal. While the majority of BC cases are sporadic, hereditary forms account for 5-10% of cases. The most common inherited mutations associated with BC are germline mutations in the BReast CAncer (BRCA) 1/2 gene (*gBRCA1/2*). They are found in approximately 5-6% of BC patients and are inherited in an autosomal dominant manner, primarily affecting younger women. Pathogenic variants within *BRCA1/2* genes elevate the risk of both breast and ovarian cancers and give rise to distinct clinical phenotypes. BRCA proteins play a key role in maintaining genome integrity by facilitating the repair of double-strand breaks through the homologous recombination (HR) pathway. Therefore, any mutation that impairs the function of BRCA proteins can result in the accumulation of DNA damage, genomic instability, and potentially contribute to cancer development and progression. Testing for *gBRCA1/2* status is relevant for treatment planning, as it can provide insights into the likely response to therapy involving platinum-based chemotherapy and poly[adenosine diphosphate (ADP)-ribose] polymerase inhibitors (PARPi). The aim of this review was to investigate the impact of HR deficiency in BC, focusing on *BRCA* mutations and their impact on the modulation of responses to platinum and PARPi therapy, and to share the experience of Unidade Local de Saúde Santa Maria in the management of metastatic BC patients with DNA damage targeted therapy, including those with the Portuguese c.156_157insAlu *BRCA2* founder mutation.

## Introduction

Breast cancer (BC) is the most frequently diagnosed malignancy among women, both in Portugal and worldwide. According to GLOBOCAN data, more than 2 million women were diagnosed with BC in 2020, and over 600,000 women are estimated to die from BC every year globally [1]. In the same year, Portugal recorded approximately 1,700 BC-related deaths [2].

Despite improvements in screening programs and perioperative systemic therapy, 25–30% of patients with early-stage BC will eventually develop metastatic disease [3], making it an incurable condition. The overall survival (OS) of patients with metastatic BC (MBC) is approximately 3 years with a 5-year OS of 25%. This survival rate depends on clinical factors such as age, performance status, and biological factors such as tumor molecular subtype, metastatic site, tumor burden, and prior therapies [4–6].

The treatment landscape for MBC has seen significant advancements in recent decades, leading to improved progression-free survival (PFS). Presently, multimodality treatment regimens allow disease control in selected patients with oligometastatic disease. The availability of new systemic agents with enhanced activity and improved safety and tolerability profiles has expanded treatment options [7, 8].

The molecular characterization of BC is important due to its association with different disease phenotypes and clinical course. Molecular classification has demonstrated both prognostic and predictive value in guiding tumor responses to chemotherapy [9]. Four main intrinsic BC subtypes have been defined (Table 1): luminal A-like, luminal B-like, human epidermal growth factor receptor 2 (HER2)-positive, and triple-negative BC (TNBC) [10].

Luminal A-like tumors are the most prevalent BC subtype, accounting for approximately 60–70% of all cases. They are characterized by the presence of high levels of estrogen receptor (ER) and/or progesterone receptor (PgR) and the absence of HER2 amplification. They are typically low grade and have low expression of the cell proliferation marker Ki-67 [11].

Luminal B-like tumors are hormone receptor positive, but may have variable degrees of ER and/or PgR expression. They are higher grade and have higher proliferation than luminal A-like tumors. They can be divided into two subgroups: “luminal B-like (HER2-negative)” and “luminal B-like (HER2-positive)”, depending on the absence or presence of HER2 amplification, respectively [12].

HER2-positive (non-luminal) BC is identified by HER2 amplification and the absence of hormone receptors, accounting for 13–15% of all cases. TNBC comprises about 15% of all BCs and is characterized by the absence of ER, PgR, and HER2 [10, 12, 13].

Luminal A tumors are linked with favorable prognosis, featuring a low incidence of relapse and high survival rates. In contrast, HER2-positive and TNBC subtypes are associated with more aggressive clinical phenotypes, often presenting with early visceral or central nervous system metastases [10, 11].

**Table 1:** Surrogate definitions of intrinsic subtypes of BC [10, 12]

**Intrinsic subtype**	**Immunohistochemical phenotype**	**Frequency (%)**
Luminal A-like	High ER and PgR; HER2-negative; low proliferation rates; typically low grade; low Ki-67 index	60–70%
Luminal B-like HER2-negative	ER-positive, but ER and PgR expression lower than in luminal A-like; HER2-negative; higher grade; high Ki-67 index	10–20%
Luminal B-like HER2-positive	ER-positive, but ER and PgR expression lower than in luminal A-like; HER2-positive; higher grade; high Ki-67 index	Both 13–15%
HER2-positive (non-luminal)	HER2-positive; ER and PgR absent; high grade; high Ki-67 index	
TNBC	ER and PgR absent, HER2-negative; higher grade; high Ki-67 index	10–15%

ER: estrogen receptor; HER2: human epidermal growth factor receptor 2; PgR: progesterone receptor; BC: breast cancer; TNBC: triple-negative breast cancer

Endocrine therapy (ET) in association with cyclin-dependent kinase 4/6 inhibitors (CDK4/6i) is the backbone of treatment for luminal MBC subtypes. In HER2-positive disease, the incorporation of anti-HER2 agents in the therapeutic armamentarium has significantly altered the disease course and prognosis. For TNBC, chemotherapy and the new antibody-drug conjugate sacituzumab govitecan represent the standard of care. More recently, the association of immunotherapy with chemotherapy has emerged as a treatment strategy in both early and advanced TNBC [13, 14].

Genetically, BC is primarily sporadic, with hereditary cases accounting for only 5–10% of occurrences [15]. Among these hereditary cases, the most common mutations involve germline mutations in the BReast CAncer (BRCA) 1/2 gene (*gBRCA1/2*), present in approximately 5−6% of patients [16]. These mutations follow an autosomal dominant inheritance pattern. It’s noteworthy that *BRCA1/2* functions as tumor suppressor genes involved in DNA repair, and mutations in these genes may predispose individuals to various human cancers. In the presence of pathogenic variants of *BRCA1* and *BRCA2*, the cumulative lifetime risk of developing BC increases to 57−65% and 45−49%, respectively. Additionally, these mutations elevate the lifetime risk of developing ovarian cancer to 39−40% for *BRCA1* mutations and 11−18% for *BRCA2* mutations.

BRCA proteins play a pivotal role in maintaining genome integrity by facilitating the repair of double-strand breaks (DSBs) through the homologous recombination (HR) pathway. Therefore, any mutation that impairs the function of BRCA proteins can result in the accumulation of DNA damage, genomic instability, and potentially contribute to cancer development and progression.

Tumors with HR deficiency exhibit increased sensitivity to therapeutic agents designed to target alternative DNA repair mechanisms compared to their HR-proficient counterparts. Consequently, therapeutic strategies targeting DNA repair have been explored in patients with *BRCA1/2* hereditary mutations.

Testing for *gBRCA1/2* mutations holds significant relevance in treatment strategy planning, as the presence of these mutations has demonstrated the potential to enhance responses to both platinum-based chemotherapy and poly[adenosine diphosphate (ADP)-ribose] polymerase inhibitors (PARPi). The outcomes of the OlympiAD (NCT02000622) and EMBRACA (NCT01945775) trials resulted in the approval of the PARPi, specifically olaparib and talazoparib, respectively, in the advanced/MBC setting [17–19].

The aim of this review was to investigate the impact of HR deficiency in BC, focusing on *BRCA* mutations and their impact on modulating responses to platinum and PARPi therapy, and to share the experience of Unidade Local de Saúde Santa Maria in the management of metastatic BC patients with DNA damage targeted therapy, including those with the Portuguese c.156_157insAlu *BRCA2* founder mutation.

## HR repair

**Figure 1:**
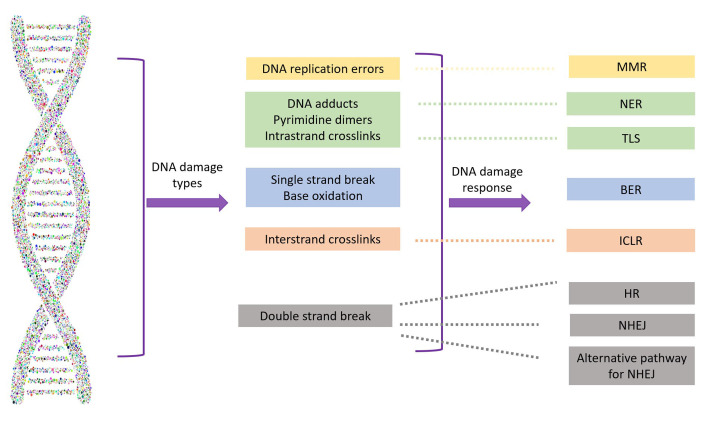
Types of DNA damage and their repair systems. MMR: mismatch repair; NER: nucleotide excision repair; TLS: translesion synthesis; BER: base excision repair; ICLR: interstrand crosslink repair; HR: homologous recombination; NHEJ: non-homologous end joining

Regardless of cell type, DNA damage driven by endogenous (e.g., reactive oxygen species) or exogenous (e.g., radiation, viruses, toxins) factors is inevitable. However, it may go unnoticed if repair mechanisms manage to correct the genetic defects. Due to the potential lethality of DNA damage, which can ultimately lead to cell death or senescence [20], a panoply of well-preserved and well-orchestrated DNA damage response (DDR) mechanisms are active at different stages of the cell cycle to preserve genome integrity. Major DDR pathways include base excision repair (BER), nucleotide excision repair, mismatch repair, non-homologous end joining (NHEJ), and HR, which is the focus of this review (Figure 1).

While single-strand breaks (SSBs) are the most common DNA insult and relatively easy to correct, DSBs pose a higher risk to genome integrity and are more difficult to manage. The preferred DDR mechanism for DSBs is NHEJ, which is efficient and generally accurate but can be error-prone. However, HR is preferred for DNA repair at replication forks, interstrand crosslinks (ICLs), or repair of programmed DSBs induced during meiosis.

HR repair (HRR) is a high-fidelity, template-dependent repair mechanism limited to the S and G2 phases of the cell cycle. In contrast to NHEJ, where DNA breaks are re-ligated, HRR copies sequences from an intact donor, usually the sister chromatid, to restore lost information, being crucial for proper genome duplication [21]. Briefly, HRR initiates with ataxia telangiectasia-mutated (ATM) and ataxia telangiectasia and Rad3-related (ATR) kinases, which recognize the DSBs and recruit other proteins involved in HRR, such as *BRCA1* and *BRCA2*, partner and localizer of *BRCA2* (PALB2), and RAD51 recombinase (RAD51). First, ATM recruits *BRCA1* to the DNA lesion site serving as the anchor point for the Mre11-Rad50-Nbs1 (MRN) complex (comprising MRE11, RAD50, and NBN). This complex facilitates the generation of 3’ single-stranded DNA (ssDNA) overhangs at the site of the break by rejecting the 5’ end. Subsequently, the 3’ ssDNA overhangs are coated by replication protein A (RPA) before *BRCA2* and PALB2 load RAD51 onto the ssDNA. This step initiates the search for homologous sequences to serve as templates for DNA extension formation, ultimately leading to the repair of the DNA break. After the displacement of the complexes, helicase- and topoisomerase-dependent cleavage or structure-selective nucleases separate the intertwined strands. The specific aspects of the HR sub-pathways involved in intertwined strand separation were reviewed by Elbakry and Löbrich [22].

### HRR-related mutations

Given that genomic instability drives tumorigenesis, it is not surprising that mutations in a variety of genes encoding proteins involved in DDR (including HRR) are frequently found in human cancers. An extensive analysis spanning various cancer types involving 500 adult patients diagnosed with metastatic solid tumors, including 91 cases of BC was conducted utilizing comprehensive whole-exome and transcriptome sequencing. This investigation revealed that potential pathogenic germline variants were detected in 12.2% of the cases, with 75% of these variants linked to deficiencies in DNA repair mechanisms [23]. Within this study, Mut Y homologue (*MUTYH*), *BRCA2*, checkpoint kinase 2 (*CHEK2*), and *BRCA1* emerged as the most frequently observed mutations. Furthermore, a comprehensive meta-analysis was conducted, spanning 189 studies and incorporating over 418,649 samples across 25 different tumor types. This comprehensive analysis specifically examined mutations in genes related to HRR genes, excluding *BRCA1/2* from consideration. This analysis demonstrated that the occurrence of mutations in HRR genes remained relatively infrequent, comprising less than 1% of cases, with ATM (5.2%), *CHEK2* (1.6%), and *PALB2* (0.9%) exhibiting the highest prevalence rates [24].

Analysis of DDR mutations in BC based on multiple studies encompassing over 5,000 cases collectively, has revealed that *ATM* and *ATR* were the most common DDR-associated genes besides *BRCA1* and *BRCA2*, with prevalence rates ranging from 2.3−4.2% and 1.3−3.7%, respectively [23, 25, 26]. Non-BRCA DDR mutations were more frequent in HER2-positive (21%) followed by luminal B (15%), TNBC (12%), and luminal A (9%) [25].

The pathological mechanisms of DDR gene mutations are well described, and based on the presence of mutations in specific genes, DDR defects can be classified into three classes. Class I includes defects in DSB and replication-associated DNA damage repair (e.g., *BRCA1*, *BRCA2*, *PALB2*, *BRIP1*, *RAD51C*, *RAD51D*); class II refers to defects in DNA damage signaling and checkpoints (e.g., *ATM*, *ATR*, *CHEK1*, *CHEK2*); and class III refers to increased mutation burden (e.g., *MSH2*, *MSH6*, *MLH1*, *PMS2*) [27]. Cancer cells with germline or somatic mutations in both alleles of genes listed in class I defects have clearly compromised HRR and in these cells, DSBs are repaired by alternative non-conservative mechanisms, such as NHEJ.

However, mutations in the different HRR genes do not necessarily result in equally severe HRR defects, and DDR- and HRR-based gene signatures have been shown to have predictive value. A combined DDR signature score (*CETN2*, *ERCC1*, *NEIL2*) was able to stratify patients who would not respond to ET but could be candidates for cyclin-dependent kinase 4/6 (CDK4/6)-based treatment [28]. Although not in BC, assays based on HRR defect signatures (which have a higher prevalence than *BRCA1/2* mutations) are currently validated and in clinical use, such as HR deficiency (HRD) detect [29, 30], My choice HRD (Myriad Genetics Inc, Sault Lake City, UT, USA) [31, 32] and Foundation Focus CDxBRCA (Foundation Medicine) [33, 34].

Overall, apart from an undeniable advantage in tumorigenesis up to a certain stage, the loss of DDR pathways in cancer cells renders them vulnerable to DNA damage. Thus, the pharmacological inhibition of DDR has become a therapeutic goal, derived from the synthetic-lethal relationships that exist between DDR genes. The dependence of cancers with *BRCA1*/*BRCA2* mutations or other HRR defects on poly[adenosine diphosphate (ADP)-ribose] polymerase (PARP) enzymes to correct DSBs leads to apoptosis in the presence of PARPi [35]. However, reactivation of HRR can occur in BRCA-mutant cells under PARPi, as determined by the presence of RAD51 foci [36, 37]. Mechanistically, replication stress induced by trapped PARP1 on DNA leads to an ATR- and *CHEK1*-orchestrated response that stabilizes replication fork structures and delays cell cycle progression until DNA repair, restart of replication fork structures, and completion of DNA synthesis prior to mitosis [38]. The following sections summarize and discuss recent advances in the therapeutic targeting of HRR in BC.

## Therapeutic approaches for DNA repair targeting

### Founder effect and Portuguese founder mutations

As mentioned earlier, *BRCA1* and *BRCA2* represent the intersection of numerous key cellular functions, engaging in multiple functions encompassing DDR and repair, chromatin remodeling, transcriptional regulation, and protein ubiquitination.

*BRCA* mutations are highly variable among populations, and many of their pathological implications remain undetermined. In Portugal, the most common *BRCA2* rearrangement is c.156_157insAlu, which originates from Portuguese ancestry and is considered a founder mutation [39].

Founder mutations have been reported in other specific populations, the best known being the Ashkenazi Jewish population (ancestry from Eastern and Central Europe) [40]. Within the context of Portuguese *BRCA* founder mutations, one extensively studied variant is the *BRCA2* c.156_157insAlu, first described in 2005 [39]. Available data suggest its origin in families from central and southern Portugal, probably 558 years ± 215 years ago. Notably, the *BRCA2* c.156_157insAlu alteration, along with two other variants within the *BRCA1* gene (c.3331_3334del and c.2037delinsCC), collectively accounts for approximately 50% of identified pathogenic mutations in the *BRCA* gene within Portuguese familial cohorts [41]. Furthermore, the observation that families bearing *BRCA1* c.3331_3334del or c.2037delinsCC mutations share a common haplotype raises the possibility that these variants may also represent founder mutations within the Portuguese population [39].

This information holds significant relevance for clinical practice because the development of mutation detection panels relies on understanding the mutation spectrum within the target population [42]. Detection of *BRCA2* c.156_157insAlu and *BRCA1* c.3331_3334del requires specific polymerase chain reaction testing, which is not universally performed by all laboratories [39].

### *BRCA*-mutated tumors and platinum therapy

**Figure 2:**
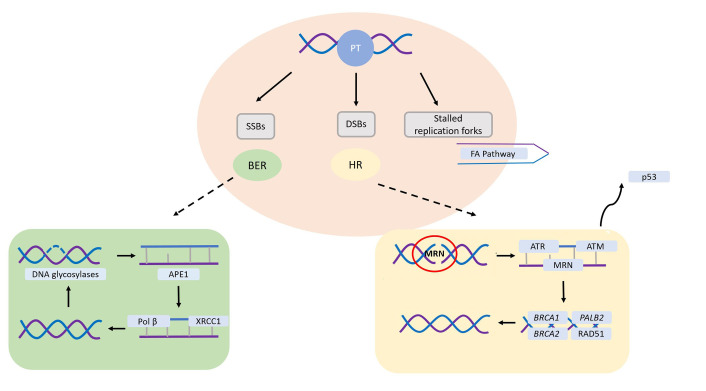
Main DNA repair pathways involved in platinum salts-induced DNA damage. SSBs are primarily repaired through the base excision repair (BER) pathway, requiring proficient DNA glycosylases to identify and cleave the damaged base. Following this, human apurinic/apyrimidinic endonuclease 1 (APE1) eliminates the abasic site, which can then be sealed by Polβ and ligases. In the instance of DSBs, HR assumes a critical role. The Mre11-Rad50-Nbs1 (MRN) complex detects the DSB, enlisting ataxia telangiectasia-mutated (ATM) and ataxia telangiectasia and Rad3-related (ATR), eventually leading to potential cell cycle arrest mediated by p53. Subsequently, ATM facilitates the recruitment of breast cancer susceptibility gene 1 (*BRCA1*), *BRCA2*, and partner and localizer of *BRCA2* (*PALB2*), determining RAD51 loading and subsequent DNA synthesis. SSBs: single-strand breaks; DSBs: double-strand breaks; HR: homologous recombination; PT: platinum salts; FA: Fanconi anaemia *Note.* Adapted from “Platinum salts in patients with breast cancer: a focus on predictive factors,” by Garutti M, Pelizzari G, Bartoletti M, Malfatti MC, Gerratana L, Tell G, et al. Int J Mol Sci. 2019;20:3390 (https://www.mdpi.com/1422-0067/20/14/3390). CC BY.

The absence of HR is a prime target for therapies inducing DSBs during the DNA replication process, where HR plays a crucial repair role. Platinum compounds have demonstrated efficacy in BC treatment, either alone or in combination therapy. They act as DNA cross-linking agents, forming intra-strand crosslinks that impede DNA synthesis, function, and transcription (Figure 2) [43]. This mechanism makes platinum therapy particularly effective for DNA repair-deficient tumors, especially those harboring mutations in *BRCA1/2* genes. Consistently, preclinical models have shown that *BRCA*-mutant cells are more sensitive to chemotherapeutic agents that cause DNA DSBs, such as platinum compounds, anthracyclines, and alkylators [44, 45].

### Platinum salts and MBC

Two key trials investigated platinum agents in advanced/metastastic BC with *gBRCA1/2* mutations: the Triple Negative Breast Cancer Trial (TNT) and the TBCRC009 trial (Table 2).

**Table 2:** Key trials of platinum agents in advanced/metastastic BC with *gBRCA1/2* mutations

**Trial**	**Population**	**Phase**	**Treatment arms**	**Outcomes**
TNT (NCT00532727) [46]	Advanced/metastatic TNBC	III	Carboplatin vs. docetaxel	Overall population: ORR 31.4% (carboplatin) vs. 34% (docetaxel); PFS 3.1 months (carboplatin) vs. 4.4 months (docetaxel); OS 3.1 months (carboplatin) vs. 4.4 months (docetaxel) *gBRCA1/2* subgroup: ORR 68% (carboplatin) vs*.* 33% (docetaxel); PFS 12.8 months (carboplatin) vs. 12 months (docetaxel)
TBCRC009 (NCT00483223) [47]	Metastatic TNBC	II	Cisplatin vs. carboplatin	Overall population: ORR 25.6% (study population); ORR 32.6% (cisplatin) vs. 18.7% (carboplatin) *gBRCA1/2* subgroup: ORR 54.6%

BC: breast cancer; *gBRCA1/2*: germline mutations in the BReast CAncer (BRCA) 1/2 gene; ORR: objective response rate; OS: overall survival; PFS: progression-free survival; TNBC: triple negative breast cancer

The TNT trial (NCT00532727) constituted a phase III trial designed to evaluate the effectiveness of carboplatin vs. docetaxel in treating TNBC [46]. The study enrolled 376 patients with recurrent locally advanced or metastatic TNBC, 43 of whom had *gBRCA1/2* mutations. The primary endpoint of the trial was the objective response rate (ORR). The study protocol included pre-specified analyses to explore interactions between biomarkers and treatment outcomes in subgroups defined by *gBRCA*-BC mutations and *BRCA-ness* characteristics, aiming to predict responses to platinum salts.

Although no significant differences in ORR, PFS, or overall survival (OS) were observed in the overall study population between the carboplatin and docetaxel arms, significantly higher ORR and PFS emerged within the subgroup possessing *gBRCA1/2* mutations. In this subgroup, the carboplatin arm demonstrated significantly higher ORR and PFS compared to the docetaxel arm (ORR 68% vs. 33%; median PFS 6.8 months vs. 4.8 months, respectively). Additionally, a significant interaction between treatment effect and *gBRCA* status was identified (*P* = 0.01) [46].

The TBCRC009 (NCT00483223) was a phase II clinical trial conducted as a single-arm study that included patients with metastatic TNBC who were administered either cisplatin or carboplatin as a first- or second-line treatment, based on investigator choice once every 3 weeks [47]. The co-primary endpoints were centered on assessing the ORR and predicting responses based on *p63/p73* gene expression levels. The ORR in the overall study population was 25.6% and was numerically higher with cisplatin (32.6%) compared to carboplatin (18.7%). Remarkably, among patients with *gBRCA1/2* mutations, the ORR increased to 54.6%, suggesting that platinum agents increase the response rate in patients with advanced or MBC [47].

## PARPi

### Mechanism of action of PARPi

Poly(ADP-ribose) polymerase (PARP) is a family of 17 proteins known for their involvement in various cellular processes, achieved through the covalent attachment of poly (ADP-ribose) chains to target molecules. This process, termed poly(ADP ribosyl)ation (PARylation), is a widespread post-translational modification at DNA lesions that is crucial for chromatin reorganization, DDR, transcriptional regulation, apoptosis, and mitosis [48, 49].

The best-studied member of the PARP family is PARP1, which is also the most highly correlated with DDR, generating nearly 90% of poly ADP-ribose chains after a DNA damage event [50]. Thus, PARP1 plays a major role in maintaining genome integrity.

Within *BRCA1/2*-mutant cells, PARPs assume a pivotal role in recognizing DNA damage and initiating alternative repair pathways. Therefore, even in the absence of functional *BRCA1/2*, these cells can persist due to the compensatory function of PARP. In this scenario, cell death occurs when there is a simultaneous loss of function in both *BRCA1/2* and PARP, a genetic concept known as synthetic lethality [51, 52]. Consequently, cells harboring *BRCA* mutations exhibit an exceptional susceptibility to the inhibition of PARP activity [37, 53].

PARPi were the first clinically approved drugs to explore the concept of synthetic lethality and represented an innovative therapeutic strategy for the treatment of tumors with *BRCA1/2* mutations [54]. PARPi structurally mimics nicotinamide adenine dinucleotide (NAD+) for the catalytic active site of PARP molecules. Physiologically, nicotinamide is the primary precursor of NAD+, an essential cofactor in the production of adenosine triphosphate (ATP) and the sole substrate of PARP1 [55]. While PARP1 is generally considered to be the primary target of PARPi, the structural resemblance of the NAD-binding domain among certain PARP family members allows PARPi to also inhibit other PARPs, such as PARP2 and PARP3. Furthermore, they may exhibit off-target effects on kinases [56, 57]. Despite these variations, various PARPi share a similar mechanism of action while displaying distinct cytotoxic profiles and potencies [58].

**Figure 3:**
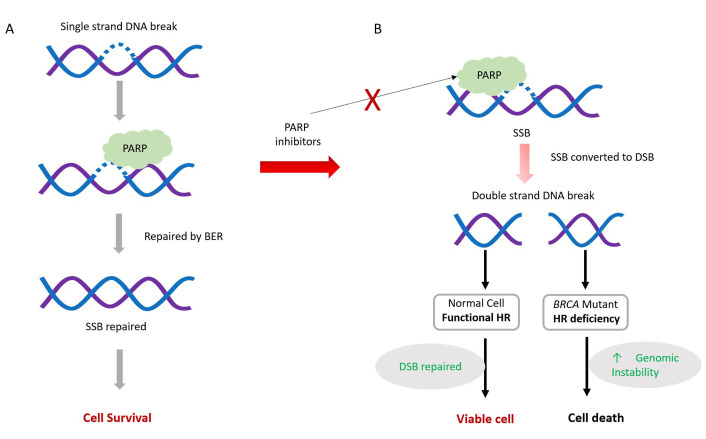
The mechanism of action of poly[adenosine diphosphate (ADP)-ribose] polymerase (PARP) inhibitors (PARPi). PARPi mechanism involves interfering with PARP’s role in repairing single-strand breaks (SSBs) present in proliferating cells. Normally, PARP assists in SSB repair, primarily through PARP-dependent base excision repair (BER), vital for cell survival (A). However, PARP inhibitors disrupt this process by preventing PARP from binding to DNA breaks. Consequently, unrepaired SSBs may evolve into double-strand breaks (DSBs), causing cellular toxicity (B). Cells proficient in homologous recombination (HR) can mend these DSBs during replication, ensuring genome stability and cell survival. Conversely, cells lacking efficient HR mechanisms fail to repair DSBs, resulting in cell apoptosis. Red ×: blocking

The mechanism of action of PARPi is not fully understood. Several mechanisms have been proposed to explain their effectiveness. These include inhibition of SSBs repair, trapping of PARP1 in DNA, and NHEJ upregulation (Figure 3).

Endogenous SSBs frequently occur in proliferating cells, and PARP proteins play a crucial role as regulators in the identification and repair of SSBs through BER. Efficient repair of SSBs is essential for cell survival. PARPi inhibits PARP activity, thereby impeding the repair of SSBs through BER.

Unrepaired SSBs can transform into DSBs, which are harmful to cells. HR is the major pathway to repair such lesions during cell replication. HR-deficient cells are unable to repair these DSBs and ultimately undergo apoptosis, eventually leading to cell death [59–61].

In the second mechanism, PARPi prevents the dissociation of PARP1 from damaged DNA, forming a DNA-protein complex that acts as a replication barrier [59, 60, 62]. Although the exact mechanism explaining the trapping of PARP1 in DNA remains unclear, two mechanisms have been proposed: (a) PARPi prevents the release of PARP1 from DNA by inhibiting auto-PARylation; or (b) PARPi binds to the catalytic site of PARP and induces changes in the enzyme’s structure, increasing its affinity for DNA [58, 63]. Since HR-deficient *BRCA*-mutated cells cannot repair DNA breaks, this leads to cell death [57, 64].

In the third mechanism, PARPi disrupts the usual inhibition of the NHEJ pathway by PARP1. Since NHEJ is error-prone, this leads to an increased number of mutations and chromosome rearrangements, and thus cell death [59, 62, 65].

Moreover, PARP1 regulates the transcription of several proteins that are crucial for cancer cell survival, including p53 and nuclear factor-kappa B (NF-κB) [66]. As a result, the use of PARPi could lead to the suppression of oncogenes controlled through PARP-dependent transcription. Additionally, PARPi downregulates nucleolar RNA helicase II (DDX21) and inhibits ribosomal DNA transcription and ribosome biogenesis in *BRCA1/2*-proficient BC, ultimately resulting in decreased cell growth [66].

### Role of PARP inhibitors in MBC

Two PARPi are currently approved for the treatment of *gBRCA1/2*-mutated MBC, based on the outcomes from the OlympiAD (NCT02000622) and EMBRACA (NCT01945775) clinical trials with olaparib and talazoparib, respectively (Table 3) [18, 19].

**Table 3:** Key trials of PARP inhibitors in advanced/MBC

**Clinical trial**	**Patient population**	**Phase**	**Treatment arms**	**Key endpoints:**
OlympiAD [67]	*gBRCA*	III	Olaparib vs. PCT	PFS 7 months (olaparib) vs. 4.2 months (placebo)
MEDIOLA [68]	*gBRCA*	I/II	Olaparib followed by durvalumab	PFS 8.2 months; ORR 63%; OS 20.5 months
EMBRACA [19]	*gBRCA*	III	Talazoparib vs. PCT	PFS 8.8 months (talazoparib) vs. 5.6 months (placebo)
JAVELIN [69]	TNBC *or gBRCA*	I/II	Talazoparib + avelumab	ORR 24.4% (*gBRCA1/2)* and 4.9% (ATM)
BROCADE 3 [70]	*gBRCA*	III	Carboplatin + paclitaxel + veliparib vs. carboplatin + paclitaxel	PFS 14.5 months (carboplatin + paclitaxel + veliparib) vs. 12.6 months (carboplatin + paclitaxel); OS 33.5 months (carboplatin + paclitaxel + veliparib) vs. 28.2 months (carboplatin + paclitaxel)
TOPACIO/Keynote-162 [71]	TNBC	II	Nirabarib + pembrolizumab	PFS (*BRCA*-mutated group) 8.1 months

*gBRCA*: germline mutations in the BReast CAncer (BRCA) gene; ORR: objective response rate; OS: overall survival; PFS: progression-free survival; PCT: physician’s choice of chemotherapy; TNBC: triple-negative breast cancer; ATM: ataxia telangiectasia-mutated

The OlympiAD trial was a randomized phase III multicenter study that enrolled 302 patients [67]. It focused on patients with *gBRCA1/2*-mutated, HER2-negative MBC who had received a maximum of two prior lines of chemotherapy for metastatic disease. The trial used a 2:1 randomization scheme, comparing olaparib monotherapy (300 mg twice daily) with physician’s choice chemotherapy (capecitabine, eribulin, or vinorelbine in 21-day cycles). The primary endpoint was PFS. At the time of the initial analysis, there was a statistically significant advantage in favor of olaparib, with a PFS of 7 months vs. 4.2 months in the standard-of-care arm. In addition, the ORR in the olaparib group was twice that of the standard of care arm (59.9% vs. 28.8%, respectively). In the first line of MBC treatment, olaparib also demonstrated a longer median OS compared with standard therapy (19.3 months vs. 17.1 months). Overall, favorable trends in outcomes were observed across all patient subgroups when treated with PARPi. Regarding side effects, grade ≥ 3 adverse events were less frequent with olaparib (36.6%) than with standard therapy (50.5%), and the rates of discontinuation due to toxicities were 4.9% and 7.7%, respectively. These compelling findings culminated in the approval of olaparib for patients with *gBRCA1/2*-mutated, HER2-negative MBC previously treated with chemotherapy in the neoadjuvant, adjuvant, or metastatic setting [18, 67].

EMBRACA was a multicenter, randomized, phase III trial that recruited a cohort of 431 patients with advanced BC and *gBRCA1/2* mutations who were randomized 2:1 to receive either talazoparib (1 mg once daily) or physician’s choice chemotherapy (capecitabine, eribulin, gemcitabine, or vinorelbine) [19]. Similar to the OlympiAD trial, the primary endpoint of median PFS favored talazoparib, with a median PFS of 8.6 months compared to 5.6 months for standard therapy. The ORR was also higher with talazoparib (62.6% vs. 27.2%). In contrast to OlympiAD, EMBRACA was powered to detect potential differences in OS. However, in the final analysis, talazoparib did not demonstrate a statistically significant improvement in OS compared to chemotherapy across clinically relevant subgroups. Grade ≥3 hematologic events occurred in 55% of patients receiving talazoparib and 38% of the patients receiving standard therapy. Based on the results of this study, talazoparib was approved for the treatment of *gBRCA1/2*-mutated, HER2-negative advanced or MBC [19].

### Overcoming resistance to PARPi

The rapid development of therapy resistance is a critical problem in cancer treatment. In the case of PARPi, the most widely accepted mechanism of therapy resistance is repairing of HR pathway by secondary mutations within the *BRCA* genes that reactivate their function. Combination therapy of PARPi with other HR-inhibiting agents (such as chemotherapy and targeted therapies) is one strategy to overcome PARPi resistance.

Combination therapy of olaparib with chemotherapy, namely with trabectidine/lurbenectidine [72, 73] and sapacitabine [73, 74] has also been investigated and showed promising results. In the next section, the combination of PARPi with immunotherapy will be reviewed.

### Combination of PARPi with immunotherapy

After preclinical models suggested that PARPi could elicit an antitumor immune response by increasing the mutagenic load [75, 76], combinations of PARPi and immunotherapy started to be investigated as a novel and promising approach in advanced BC. Several clinical trials investigating combinations of PARPi with immunotherapy in advanced BC are currently ongoing or have already been completed and shown results (Table 3).

The phase I/II MEDIOLA trial (NCT02734004) evaluated the effectiveness of olaparib in combination with the anti-programmed death-ligand 1 (PD-L1) durvalumab in patients with solid tumors [68]. Initially, four cohorts were enrolled, with all patients having received a maximum of two prior lines of chemotherapy. Among these cohorts, a subgroup of 30 patients had HER2-negative, *gBRCA1/2*-mutated MBC. Patients were treated with olaparib (300 mg twice daily) for an initial 4 weeks, followed by a combination of olaparib (300 mg twice daily) and durvalumab (1.5 g every 4 weeks) until disease progression occurred. The primary endpoints were safety and disease control rate (DCR) at 12 weeks. The 12-week DCR was 80%, with a median OS of 21.5 months and an ORR of 63%, comparable to findings reported in the OlympiAD trial. The combination was generally well-tolerated with no observed increase in immune-related adverse events [68].

The JAVELIN Solid Tumor trial (NCT01772004) was conducted in phases I/II and aimed to assess the efficacy of talazoparib combined with the anti-PD-L1 agent avelumab in patients with *gBRCA1/2*- or *ATM*-altered advanced or metastatic solid tumors [69]. Patients received avelumab (800 mg every 2 weeks) and talazoparib (1 mg daily) until disease progression or unacceptable toxicity. The primary outcome measure was ORR according to RECIST 1.1 criteria, and preliminary results showed an ORR of 24.4% in the *gBRCA1/2* cohort and 4.9% in the ATM cohort [69].

Niraparib, another highly selective PARP1/2 inhibitor, has shown activity in advanced or metastatic TNBC. The TOPACIO/KEYNOTE-162 trial (NCT02657889) was a single-arm phase II study of niraparib in combination with immunotherapy for patients with advanced/metastatic TNBC, regardless of *BRCA* mutation status or PD-L1 expression [71]. Patients received oral niraparib (200 mg once daily) in combination with pembrolizumab (200 mg intravenously on day 1 of each 21-day cycle). The primary endpoint was ORR, while the secondary endpoint was DCR. Among the 55 women in the global study population, five achieved confirmed complete responses, while another five achieved confirmed partial responses. Additionally, 13 patients exhibited stable disease, and 24 experienced progressive disease. In the efficacy-evaluable population (*n* = 47), the ORR (*n* = 10) was 21% and the DCR (*n* = 23) was 49%. Notably, among the 15 evaluable patients with *BRCA* mutations, seven achieved an ORR of 47%, twelve attained a DCR of 80%, and PFS was 8.3 months [71].

### Other PARPi in development in advanced/MBC

In addition to the currently approved PARPi olaparib and talazoparib, ongoing research is exploring other agents for the treatment of BRCA-mutated advanced/MBC. Among these, veliparib and niraparib are currently the most promising, in both advanced and localized disease settings.

Veliparib is designed to specifically inhibit PARP1 and PARP2. Notably, it appears to have less PARP trapping potential, making it an appealing option for use in combination therapy alongside conventional chemotherapy agents (Table 3) [70, 77].

The phase III, placebo-controlled BROCADE 3 trial (NCT02163694) enrolled 513 patients with *gBRCA1/2*-mutations, HER2-negative BC. These patients had received a maximum of two prior lines of chemotherapy for metastatic disease [70]. In this trial, patients were randomized in a 2:1 scheme to receive carboplatin on day 1 and paclitaxel (80 mg/m^2^ intravenously) on day 1, day 8, and day 15 of 21-day cycles. These chemotherapy combinations were either paired with veliparib (120 mg orally twice daily on day 2 to day 5) or a matching placebo. Patients who discontinued carboplatin and paclitaxel before progression had the option to continue receiving veliparib or placebo at an intensified dose (300 mg twice daily continuously, escalating to 400 mg twice daily if tolerated) until disease progression. The primary endpoint was PFS. After a median follow-up of 35.7 months in the veliparib group and 35.7 months in the control group, PFS was reported as 14.5 months for the velaparib group and 12.6 months for the control arm. Grade ≥ 3 events were more prevalent in the veliparib group (34% vs. 29% in the control group [70].

It should be noted that there is currently no evidence to support the efficacy of platinum agents after PARPi (or vice versa) in the advanced/MBC setting. In addition, at the time of this review, there were no data comparing PARPi and platinum agents.

## Current development of other DNA damage-targeted treatments for BC

Currently, drugs targeting other players involved in DNA damage, such as ATR, ATM, CHEK1/2, and WEE1, are being investigated for their clinical potential.

### ATR inhibitors

ATR kinase belongs to the phosphatidylinositol 3-kinase-related kinase (PIKK) family and functions as the primary responders to single-strand DNA damage, playing a crucial role as master regulators of replication stress [78].

An innovative oral inhibitor targeting ATR kinase, RP-3500 (camonsertib), is being tested in the NCT04497116 trial. This ataxia telangiectasia and Rad3-related kinase inhibitor (ATRi) exhibited efficacy in cellular biochemical assays, significant selectivity for ATR, and substantial potential for reducing tumor growth in murine models when orally administered once daily at doses ranging between 5–7 mg/kg [79].

When combined with olaparib in short-term treatments, RP-3500 displayed a synergistic effect surpassing that of sequential administration, exceeding the individual efficacy of olaparib and being more potent at lower doses. This simultaneous treatment also excelled at inducing tumor cell death compared to continuous treatment, especially promising for BC with DRR [79].

Another orally administered ATRi, AZD6738 (ceralasertib), exhibited notable selectivity in inhibiting ATR kinase [80]. AZD6738 has a combined therapeutic effect with agents known for causing replication fork stalling and collapse, such as carboplatin, irinotecan, and olaparib. Antitumor therapy is achieved synergistically at lower doses compared to using each agent alone. This suggests a potential strategy for enhancing therapeutic effects while minimizing individual drug dosages [80].

In a model using TNBC xenografts derived from patients with a *BRCA2* mutation, complete regression of tumors was observed within 3 days to 5 days. This regression occurred when AZD6738 was administered in conjunction with olaparib daily, three times to five times a week. Moreover, escalating the dosage of olaparib or increasing the frequency of AZD6738 administration to twice daily resulted in complete tumor regression, even in a TNBC xenograft model without the *BRCA* mutation [80].

Berzosertib (previously known as VX-970) is a potent and specific suppressor of ATR, demonstrating preclinical anticancer activity in combination with DNA-damaging chemotherapy in TNBC [81, 82]. In an ongoing C2 expansion trial (phase 1b trial) investigating safety, tolerability, efficacy, and potential predictive biomarkers of berzosertib in combination with cisplatin among patients with advanced TNBC (specifically those with tumors characterized as *gBRCA1/2* wild-type and basal subtype) berzosertib was well tolerated [82]. The ORR was 23.4% [90% confidence interval (CI): 13.7, 35.8]. No relevant associations were observed between the response and gene alterations. Further studies combining ATR inhibitors with platinum compounds may be warranted in highly selected patient populations [81, 82].

### CHEK1/2 inhibitors

CHEK1/2 serves as downstream targets of both ATR and ATM, playing a crucial role in the temporary arrest of the cell cycle and the repair of DNA damage [83]. In a phase II single-arm study (NCT02203513), the efficacy of prexasertib at 105 mg/m^2^ IV every 2 weeks was assessed in patients with metastatic/recurrent TNBC. The primary endpoint was ORR. This study enrolled 9 patients with *gBRCA* wild type who had undergone at least one prior treatment. Prexasertib demonstrated modest clinical efficacy in *BRCA*-wild type TNBC, with one patient experiencing a partial response (ORR 11.1%) and four patients achieving stable disease [84].

One promising therapeutic strategy involves combining prexasertib with PARPi, since it has been shown that when olaparib is combined with a CHEK1 inhibitor, it diminishes HR efficiency and reduces replication fork stability [85].

In a phase I study where prexasertib was combined with olaparib in high-grade serous ovarian cancer (NCT03057145), preliminary clinical activity was observed specifically in *BRCA*-mutant patients who had previously progressed on a PARPi. Pharmacodynamic analyses revealed that prexasertib compromises HR, inducing DNA damage and replication stress [86]. This insight can provide future guidance for combining PARPi and other DDR-target therapies.

### Wee inhibitors

Wee1-like protein kinase (WEE1) regulates the intra-S and G2/M cell-cycle checkpoints [87]. Adavosertib (MK-1775) is a highly effective and specific inhibitor of WEE1, inducing DNA damage regardless of any adjuvant chemotherapy or radiotherapy.

Promising results were observed in phase II trials when adavosertib was combined with gemcitabine for patients with platinum-resistant or refractory ovarian cancer (NCT02151292). In this study, the adavosertib group exhibited a prolonged PFS compared to the placebo group (4.6 months vs. 3.0 months), but with higher rates of neutropenia and trombocitopenia [88].

In another phase II trial (NCT01357161) that enrolled women with histologically or cytologically confirmed epithelial ovarian, fallopian tube, or peritoneal cancer with measurable disease, patients were administered adavosertib (oral capsules, with 2 days on/5 days off or 3 days on/4 days off schedules) across six cohorts ranging from 175 mg once daily to 225 mg twice daily. These were combined with gemcitabine, paclitaxel, carboplatin, or pegylated liposomal doxorubicin. The primary outcome measurement was ORR. Three percent of patients achieved a confirmed complete response, and 29% achieved a confirmed partial response. The response rate was notably highest (66.7%) with carboplatin plus weekly adavosertib, showing a 100% DCR and a median PFS of 12.0 months. The longest median duration of response was observed in the paclitaxel cohort (12.0 months) [89]. Despite the potential of combining PARPi and WEE inhibitors in trapping tumor progression, this combination is poorly tolerated [90].

## Experience of Unidade Local de Saúde Santa Maria

At Unidade Local de Saúde (ULS) Santa Maria, the criteria for testing for g*BRCA 1/2* mutations include one of the following: BC diagnosed at ≤ 45 years of age; bilateral/synchronous tumors (provided they are clearly distinct) or homolateral metachronous tumors, both in subjects ≤ 60 years of age; TNBC histology, regardless of age; BC diagnosed in male patients; or diagnosis of MBC in any patient with a genetic study result that impacts the therapeutic decision (e.g., eligibility for PARPi therapy).

Five patients with MBC and *BRCA1/2* mutations were identified and treated with platinum and/or PARPi therapy between May 2018 and December 2022 at our Oncology Department (Table 4). All patients were female, with a median age of 39.4 years (range 29−57 years). Histological analysis revealed that all tumors were invasive carcinomas of no special type (NST), and three had grade 2 differentiation. Regarding the molecular subtype, four patients had luminal B/HER2-negative BC and one had TNBC. At diagnosis, three patients were in stage IV and two were in stage II. The most common metastatic site at MBC diagnosis was bone (*n* = 4). Four patients had *BRCA2* mutations, and one had a *BRCA1* mutation.

**Table 4:** Population, tumor, and treatment characteristics of the MBC cohort of ULS Santa Maria

**Variable**			**Value**
Demographic characteristic	Mean age (years)	-	39.4 (range 29–57)
	Gender (female)	-	100% (*n* = 5)
*BRCA* mutations (%, *n* patients)	*BRCA1*	-	20% (*n* = 1)
	*BRCA2*	-	80% (*n* = 4)
Tumor characteristic (%, *n* patients)	Histologic subtype	NST	100% (*n* = 5)
	Molecular subtype	Luminal B HER2-negative	80% (*n* = 4)
		TNBC	20% (*n* = 1)
	Differentiation grade	2	60% (*n* = 3)
		3	40% (*n* = 2)
	Stage	II	40% (*n* = 2)
		IV	60% (*n* = 3)
	PARPi [80% (*n* = 4)]	Olaparib	20% (*n* = 1)
		Talazoparib	60% (*n* = 3)
	Platinum	-	30% (*n* = 2)
Line of treatment (PARPi therapy; %, *n* patients)	Second line	-	40% (*n* = 2)
	Third line	-	20% (*n* = 1)
	Fourth line	-	20% (*n* = 1)

HER2: human epidermal growth factor receptor 2; NST: no special type; PARPi: poly[adenosine diphosphate (ADP)-ribose] polymerase inhibitor; TNBC: triple negative breast cancer; *BRCA*: BReast CAncer gene; HER2: human epidermal growth factor receptor 2; -: no data

**Figure 4:**
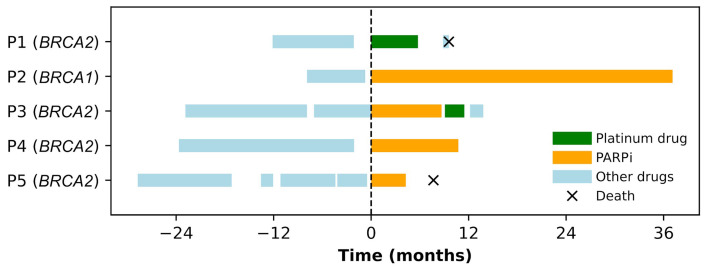
Swimmers plot for metastatic breast cancer (BC) susceptibility gene 1/2 (*BRCA1/2*)-mutated BC patients treated with platinum drugs or poly[adenosine diphosphate (ADP)-ribose] polymerase inhibitor (PARPi) at Unidade Local de Saúde Santa Maria between May 2018 and June 2022. Each continuous horizontal line represents a line of treatment for metastatic disease. The origin of the X-axis is taken as the starting date of the first time that a platinum drug or PARPi is used in a metastatic setting. Negative time points represent the number of months before the use of the first PARPi or platinum compound, while positive time points represent the number of months since the use of the first PARPi or platinum compound. P: patient

Two patients [patient 1 (P1) and P3, Figure 4] received treatment with platinum agents, including carboplatin. Both were platinum-naive and received this therapy as a fourth-line treatment for MBC.

Four patients received PARPi, one patient received olaparib as the second-line for metastatic disease, and three patients received talazoparib in the second (*n* = 1), third (*n* = 1), or fourth (*n* = 1) line for metastatic disease. One patient (P3) received PARPi and platinum in the third and fourth lines of MBC therapy, respectively. Platinum was administered after progression with a visceral crisis.

In this small cohort, the PFS for patients receiving platinum agents was 5.8 months for P1 and 2.4 months for P3. The PFS for patients progressing under PARPi was 8.7 months for P3 and 4.3 months for P5. P2 and P4 have shown an ongoing response to PARPi after a follow-up of 37.1 months and 10.7 months, respectively, both displaying stable disease as their best response (Figure 4).

## Conclusions

Conventional chemotherapy despite its systemic nature and lack of the ability to discriminate between malignant and non-malignant cells remains the standard of care for treating numerous cancer types.

Cancer treatment research is advancing to identify strategies that not only effectively combat cancer but also enhance patients’ quality of life by striking a balance between efficacy and reducing treatment-related toxicity. One such approach involves the use of targeted therapies that specifically target cancer cells based on particular mutations or abnormal expression patterns. These therapies aim to effectively inhibit tumor growth and progression while minimizing their impact on non-malignant cells. Due to their targeted nature, these therapies are less likely to cause off-target side effects, leading to more favorable patient outcomes.

The role of targeting DNA repair pathways in patients with MBC and *BRCA1/2* mutations is well established. Researchers are exploring combination treatment regimens as an additional strategy to enhance treatment efficacy and reduce the development of resistance in these tumors. This approach holds promise for improving the overall management of MBC in patients with *BRCA1/2*.

Testing for *BRCA* mutations plays a crucial role in treatment planning, as the most commonly recognized alterations that confer sensitivity to PARPi are loss-of-function mutations in the *BRCA1* and *BRCA2* genes [91].

However, it has been suggested that patients with tumors with HRD due to mutations in other genes within the HR pathway may benefit from PARPi [92]. In the MBC setting, patients harboring PALB2 mutations have shown positive responses to PARPi, as well as those with *ATM* or *CHEK2* mutations [93].

Molecular profiling of *BRCA 1/2* and additional HR-related genes can be performed on blood for germline testing or on tissue for both germline and somatic testing. Blood tests exclusively identify germline mutations and are not suitable for concurrent analysis of the HRD phenotype in both germline and somatic contexts. In addition, it should be noted that the interpretation of HR-related gene mutations remains a complex clinical challenge. The decision for (germline or somatic) testing should take into account the specific PARPi approval [94, 95].

In the future, the identification of reliable biomarkers and the development of clinical trials including patients with somatic *BRCA* mutations are warranted to improve the treatment and outcomes of patients with advanced/MBC.

## Abbreviations

ADP: adenosine diphosphate
ATM: ataxia telangiectasia-mutated
ATR: ataxia telangiectasia and Rad3-related
BC: breast cancer
BER: base excision repair
BRCA: BReast CAncer gene
CHEK2: checkpoint kinase 2
DCR: disease control rate
DDR: DNA damage response
DSBs: double-strand breaks
ER: estrogen receptor
gBRCA1/2: germline mutations in the BReast CAncer 1/2 gene
HER2: human epidermal growth factor receptor 2
HR: homologous recombination
HRD: homologous recombination deficiency
HRR: homologous recombination repair
MBC: metastatic breast cancer
NAD: nicotinamide adenine dinucleotide
NHEJ: non-homologous end joining
ORR: objective response rate
OS: overall survival
PALB2: partner and localizer of BReast CAncer gene 2
PARP: poly[adenosine diphosphate-ribose] polymerase
PARPi: poly[adenosine diphosphate-ribose] polymerase inhibitors
PD-L1: programmed death-ligand 1
PFS: progression-free survival
PgR: progesterone receptor
SSBs: single-strand breaks
ssDNA: single-stranded DNA
TNBC: triple-negative BC

## Declarations

### Availability of data and materials

The raw data supporting the conclusions of this manuscript will be made available by the authors (Vanessa Patel, vanessacpatel@gmail.com) , without undue reservation, to any qualified researcher.

### Copyright

© The authors 2024.
